# In vitro study of the ecotoxicological risk of methylisothiazolinone and chloroxylenol towards soil bacteria

**DOI:** 10.1038/s41598-022-22981-9

**Published:** 2022-11-09

**Authors:** Marta Nowak-Lange, Katarzyna Niedziałkowska, Przemysław Bernat, Katarzyna Lisowska

**Affiliations:** Department of Industrial Microbiology and Biotechnology, Faculty of Biology and Environmental Protection, University of Lodz, 12/16 Banacha Street, 90-237 Lodz, Poland

**Keywords:** Environmental biotechnology, Environmental microbiology, Microbiology techniques

## Abstract

Methylisothiazolinone (MIT) and chloroxylenol (PCMX) are popular disinfectants often used in personal care products (PCPs). The unregulated discharge of these micropollutants into the environment, as well as the use of sewage sludge as fertilizer and reclaimed water in agriculture, poses a serious threat to ecosystems. However, research into their ecotoxicity towards nontarget organisms is very limited. In the present study, for the first time, the ecotoxicity of biocides to *Pseudomonas putida, Pseudomonas moorei, Sphingomonas mali*, and *Bacillus subtilis* was examined. The toxicity of MIT and PCMX was evaluated using the microdilution method, and their influence on the viability of bacterial cells was investigated by the AlamarBlue® test. The ability of the tested bacteria to form biofilms was examined by a microtiter plate assay. Intracellular reactive oxygen species (ROS) production was measured with CM-H2DCFDA. The effect of MIT and PCMX on phytohormone indole-3-acetic acid (IAA) production was determined by spectrophotometry and LC‒MS/MS techniques. The permeability of bacterial cell membranes was studied using the SYTOX Green assay. Changes in the phospholipid profile were analysed using LC‒MS/MS. The minimal inhibitory concentrations (MICs) values ranged from 3.907 to 15.625 mg L^−1^ for MIT and 62.5 to 250 mg L^−1^ for PCMX, indicating that MIT was more toxic. With increasing concentrations of MIT and PCMX, the cell viability, biofilm formation ability and phytohormone synthesis were maximally inhibited. Moreover, the growth of bacterial cell membrane permeability and a significantly increased content of ROS were observed, indicating that the exposure caused serious oxidative stress and homeostasis disorders. Additionally, modifications in the phospholipid profile were observed in response to the presence of sublethal concentrations of the chemicals. These results prove that the environmental threat posed by MIT and PCMX must be carefully monitored, especially as their use in PCPs is still growing.

## Introduction

Currently, much attention is given to the pollution of the natural environment with toxic compounds of anthropogenic origin. Mass production and application of synthetic chemicals increase this threat. One of the groups of pollutants is micropollutants, which are not covered by existing statutes due to their low accumulation in the environment (i.e., ng L^−1^ up to µg L^−1^). These contaminants have been introduced into ecosystems for many years, but current advances in analytical processes have allowed the detection of their presence (despite their low quantities in the environment)^1^. Among these micropollutants, personal care products (PCPs) and household chemicals are important emerging contaminants. PCPs form an integral part of the daily lives of humans and include skincare products, hair care formulations, toothpastes, soaps, sunscreens and perfumes. These chemicals are pervasive in the environment due to their incomplete elimination by conventional biological wastewater treatment systems. Although most PCPs are considered readily biodegradable in environmental matrices, their threat to the environment is not due to persistency but rather to their biological activity together with their continuous emission, which characterizes them as “emerging contaminants” or “prospective pollutants”^2,3^.

Chloroxylenol (PCMX, 4-chloro-3,5-dimethylphenol, p-chloro-m-xylenol) and methylisothiazolinone (MIT, 2-methyl-4-isothiazolin-3-one) are broad-spectrum antimicrobial agents that are used extensively in industrial, consumer and health care products, including cosmetics, household chemicals and disinfection products such as preservatives or disinfectant agents^4,5^. PCMX is a to strong antimicrobial capable of reducing populations of bacteria, such as *Pseudomonas aeruginosa, Escherichia coli, Proteus vulgaris,* and *Salmonella typhi*, and fungi, such as *Aspergillus niger*, *Aspergillus flavus*, *Candida albicans* and *Candida parapsilosis*. Moreover, the virucidal activity of PCMX towards Ebola virus and severe acute respiratory syndrome coronavirus 2 (SARS-CoV-2) has been confirmed, making it widely used for disinfection during the SARS-CoV-2 pandemic^6–11^. Generally, it is assumed that the mechanism of action of chloroxylenol is similar to that of other phenolic and halophenolic antibacterial agents, particularly those perturbing cell membranes and causing cell leakage^4,12^. MIT, which is the best representative of preservatives from the isothiazolinones group, is a powerful biocide and has been described to be able to diffuse across the bacterial cell membrane and the cell wall of fungi. The main mechanism of the inhibitory effect is based on the presence of a reducing sulfur in the MIT molecule. Sulfur is able to react with nucleophilic groups in cellular components and inactivate thiols in cellular proteins to form disulfide bonds (-S-S-). By blocking several specific enzymes, isothiazolinones effectively stop respiration and inhibit the synthesis and utilization of adenosine triphosphate (ATP), which inhibits cellular activity and ultimately causes bacterial and fungal cell death. Moreover, ROS accumulate because the vital pathways in cellular metabolism are disrupted; this accumulation leads to cellular death^13–17^.

Considering the large presence of MIT and PCMX in everyday products, their entry into water or soil systems is inevitable, and their presence may lead to alterations in the edaphic environment. Moreover, because popular antimicrobial agents such as triclosan and triclocarban have been banned by the Food and Drug Administration of the United States, it is anticipated that alternative chemicals, such as MIT and PCMX, which are commonly perceived as less harmful, will be used in PCPs in higher quantities. Therefore, the concentrations of MIT and PCMX in the natural environment should be continually surveyed^18^. The occurrence and fate of MIT and PCMX in wastewater treatment plant (WWTP) systems have frequently been detected at the level of nano- and micrograms per litre^19–25^. Discharge of wastewater after incomplete removal of preservatives from WWTPs leads to the accumulation of residue in the receiving environment. PCMX was detected in the range of 20–1200 ng L^−1^ in surface waters in Jakarta city^26,27^. There are few data in the literature about the occurrence of MIT in surface water. Paijens et al.^28^ describes the contamination of Paris wastewater by MIT at a concentration of 14 ng L^−1^.

Because preservatives, such as MIT and PCMX, are designed to elicit biological effects, the potential exists for these compounds to affect nontarget organisms. The toxic effects of MIT and PCMX have been explored previously, mainly on aquatic organisms. In zebrafish and rainbow trout, prolonged PCMX and MIT exposure cause hatching delays or inhibition, altered gene expression, embryonic mortality, morphological abnormalities, neurotoxicity and DNA damage to erythrocytes^29,30^. Moreover, Lee et al.^31^ revealed that exposure to MIT caused modulation of genes involved in thyroid hormone regulation. PCMX also modulated antioxidant enzymatic activity, produced changes in swimming speed and caused neurotoxic and mitochondrial malfunction in the estuarine rotifer *Brachionuskoreanus*^32^.

The possible influence of PCPs on terrestrial fauna, especially soil-dwelling organisms, has not been adequately examined. Soil is frequently the final reservoir for most pollutants that penetrate the environment. The application of sewage sludge or the use of reclaimed water as an important water resource in the field is becoming more controversial. A great benefit of this practice is the conversion of precious components such as plant nutrients and organic matter. However, biosolids and recycled water, which are sources of harmful xenobiotics, can reach agricultural lands, and the chemicals can persist in soil for prolonged periods of time^33,34^. Moreover, new data in the literature suggest that, due to their biocidal activity, preservatives from the isothiazolinone group could be used as a new class of insecticides for the control of pests, which makes them a new source of environmental contamination^35^. Literature data show that MIT is present in soil in Poland at concentrations ranging from 1.04 to 10.8 µg kg^−1^^24^. Soils work as a large bioreactor for degrading pollutants and simplifying nutrient transformation. Moreover, soils play a vital role in ecosystems as a habitat for organisms and plants. Microorganisms are one type of living form that are abundant in soil and are referred to as soil microbiomes. Soil contains a wide variety of microorganisms, including bacteria, archaea, fungi, algae, and nematodes, that can be beneficial or pathogenic. Beneficial microbes in soil are essential for maintaining soil ecosystem health by breaking down organic matter, recycling elements, and promoting plant growth^36^. Among the variably distributed heterotrophic microflora, populations of bacteria belonging to different species make up approximately 15% of the overall microbial populations^37^. Undoubtedly, soil is a notable sink antimicrobial agents, which has a negative impact on indigenous microbes by killing specific groups of soil microbial flora^38,39^. Since it is known that MIT and PCMX have antibacterial activity, they could be expected to exert deleterious effects on bacteria in the soil. The antibacterial effects of MIT and PCMX have been primarily studied on pure cultures of human pathogenic bacteria^40–43^. Similar to other xenobiotics, the negative impact of these preservatives on environmentally significant bacteria is gradually increasing, but it is still poorly investigated.

The present study, for the first time, investigated the ecotoxicity of these two preservatives on species belonging to the three different genera *Pseudomonas*, *Sphingomonas* and *Bacillus*, which are representative genera of beneficial soil bacteria. The species belonging to these genera are well known for their multifaceted functions ranging from producing highly beneficial phytohormones, such as indole acetic acid, gibberellins and sphingan, to remediating many types of environmental contamination. These microbes have also been noted to reduce stress factors, such as salinity, heavy metals, and drougth, leading to improved plant growth in agricultural soil^37,44,45^.

This study was designed to investigate the tolerance of the bacterial species *Pseudomonas putida* (DSM 291), *Pseudomonas moorei* (DSM 12647), *Sphingomonas mali* (DSM 10565), and *Bacillus subtilis* (DSM 3657) to MIT and PCMX, reactive oxygen species production, changes in the phospholipid profile, biofilm formation and indole-3 acetic acid production. The objectives of the present work were to gain insight into the toxic effects of MIT and PCMX on beneficial soil bacteria and to evaluate the ecological risks they pose to soil environments.

## Results and discussion

### Determination of bacterial sensitivity and viability towards MIT and PCMX

**Figure 1:**
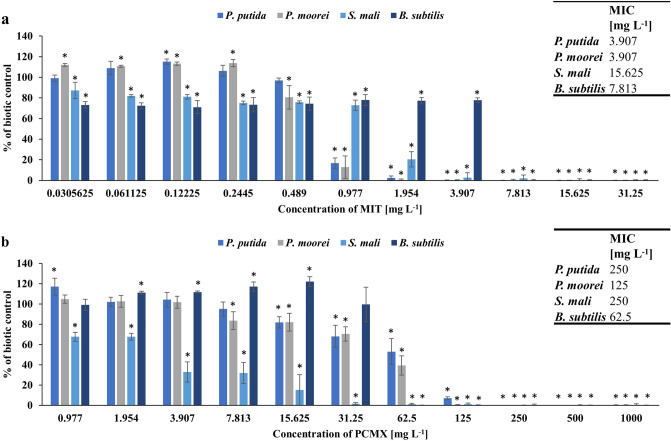
The growth of the tested strains incubated with the addition of methylisothiazolinone (**a**) or chloroxylenol (**b**). Data are expressed as the average percentage of bacterial growth (compared to biotic control) ± SD (n = 8). The presented results were analysed using the Mann‒Whitney U test with *p < 0.05.

The rich diversity and abundance of soil microflora and their activity determine the good quality and fertility of the soil. Although chemicals such as antibiotics, personal care products and plant protection products are an integral part of human life, their impact on nontarget organisms poses a serious threat to the functioning of the entire environment, including soil ecosystems. Disrupting the number or ratio of microbes in the soil can potentially inhibit the recycling and transformation processes of elements or pollutants. Studies on biocide toxicity on soil bacteria and other microorganisms are limited and generally focused on biodegradation of these pollutants or antibacterial activity in the context of using them in the final consumer products^46–48^. In the present study, for the first time, the ecotoxicology potential of MIT and PCMX has been evaluated towards gram-negative *P. putida*, *P. moorei*, and *S. mali* and gram-positive *B. subtilis* bacterial strains by determining their growth after treatment with various concentrations of MIT and PCMX. The results are presented as the percent of biotic control growth (control of bacterial growth without the addition of xenobiotics). Moreover, based on the level of bacterial growth, the MIC was measured. The effects of MIT and PCMX concentrations on bacterial growth and MIC values are illustrated in Fig. 1. The tested preservatives showed differential antimicrobial activity. For the first time, it was observed that the MIC values of preservatives towards soil microorganisms ranged from 3.907 to 15.625 mg L^−1^ for MIT and from 62.5 to 250 mg L^−1^ for PCMX, which clearly indicated that MIT was the most ecotoxic compound. The incubation of *P. putida* with MIT or PCMX at concentrations of 0.12225 mg L^−1^ and 0.977 mg L^−1^, respectively, caused statistically significant stimulation of growth. A similar effect was observed for *P. moorei* after incubation with MIT (concentration range of 0.0305625–0.2445 mg L^−1^) and *B. subtilis* after incubation with PCMX (concentration range of 1.954–15.625 mg L^−1^) with p < 0.05. It is known that some beneficial soil bacteria, such as *Pseudomonas* sp. and *Bacillus* sp., which are characterized as plant growth-promoting rhizobacteria (PGPR), can degrade various contaminants in soil and use them as a source of energy that stimulates bacterial growth^49,50^. Thus, it could not be excluded that MIT and PCMX, in low concentration ranges, are used as sources of carbon and energy.

Both strains of *Pseudomonas* were similarly susceptible to the action of MIT and PCMX. The first significant inhibition of growth was observed at an MIT concentration of 0.977 mg L^−1^, which caused approximately 80% growth inhibition of these bacteria. A further increase in the concentration led to a complete inhibition of growth. In the case of PCMX, an increased sensitivity of *Pseudomonas* was observed at concentrations above 31.25 mg L^−1^, where the xenobiotic reduced bacterial growth by more than 50% and 60% for *P. putida* and *P. moorei*, respectively. *S. mali* and *B. subtilis* were the most resistant microorganisms to the tested concentrations of MIT. In both cases, the concentration of 0.977 mg L^−1^ inhibited bacterial growth by approximately 23%. However, in contrast to that of *B. subtilis*, *S. mali* growth was highly reduced at the next tested concentration (1.954 mg L^−1^).

**Figure 2:**
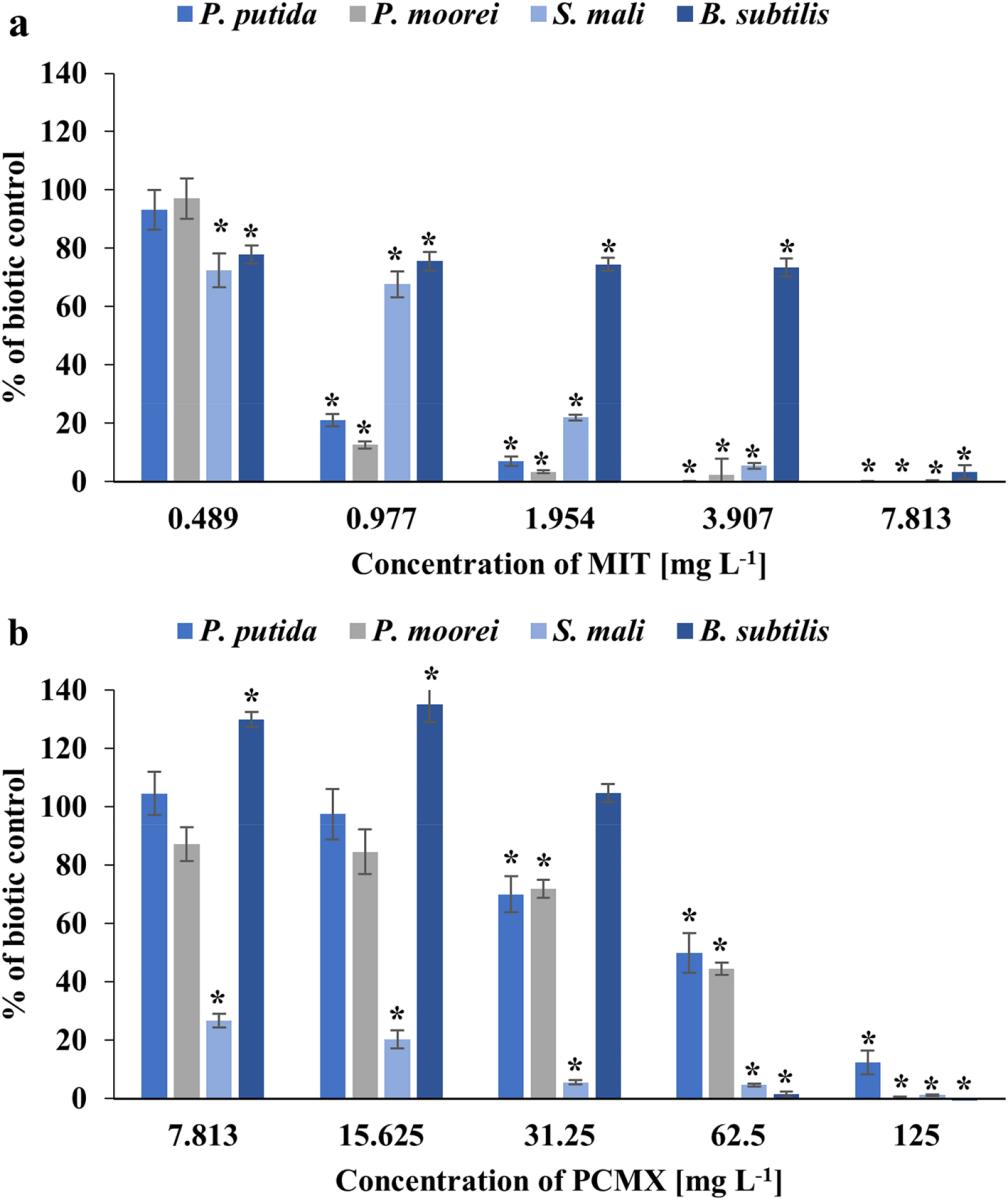
Reduction of resazurin by the tested strains incubated with the addition of methylisothiazolinone (**a**) or chloroxylenol (**b**). Data are expressed as the average percentage of bacterial growth (compared to biotic control) ± SD (n = 4). The presented results were analysed using the Mann‒Whitney U test with *p < 0.05.

The toxicity of the investigated compounds, at five significant concentrations, towards bacterial species was also examined using an AlamarBlue® (AB) assay. The AB assay is widely applied in studies for monitoring microbial viability based on measuring the difference in the fluorescence intensity of the nonfluorescent, blue dye resazurin and the highly fluorescent reduced form resofurin. This redox reaction is a result of a metabolic pathway and cell respiration of metabolically active bacteria^51,52^. The results are presented as the percent of biotic control. The results presented in Fig. 2 indicate that the inhibition of AB dye reduction could be highly correlated with the inhibition of bacterial species growth, which might suggest that the inhibition of cell growth is caused by the inhibition of metabolic activity by xenobiotics.

There are no available studies on the ecotoxicity of MIT and PCMX towards beneficial soil bacteria. Moreover, literature data about the influence of other personal care products on soil bacteria are very scarce. The antibacterial activity of the described micropollutants was evaluated mainly for microorganisms being the target of treatment. Despite the fact that these preservatives are very popular in the chemical industry, there are no recent studies describing their antimicrobial activity. For instance, Collier et al.^16^ evaluated the antimicrobial activity of compounds belonging to the isothiazolinone group towards *Schizosaccharomyces pombe* and *Escherichia coli*. MIC values for MIT achieved 245 mg L^−1^ for gram-negative rods and 41 mg L^−1^ for yeast. Lear et al.^53^ evaluated the MIC for PCMX towards industrial strains of *Pseudomonas* sp. The obtained values ranged from 200 mg L^−1^ to even above 1000 mg L^−1^. Although those studies were conducted on industrial or clinical strains, their results are similar to those obtained in our work. The differential tolerance of the tested bacteria to biocides can lead to a shift in the bacterial community composition and variation in the total number of bacteria. Several studies have indicated that the presence of some pharmaceuticals and personal care products in agricultural soil, at concentrations exceeding the tolerance of microorganisms, can reduce the diversity of the microbial community and completely change its structure. Such changes could disturb the proper functioning and health of soil^34,54,55^.

### Inhibition of biofilm formation

According to the study by^56,57^, biofilm formation by beneficial soil bacteria plays an important role in plant health. Bacterial biofilms on the surface of plants and soil can stimulate plant growth and protect against pathogenic microorganisms and stressful environmental conditions, such as contamination by pesticides, antibiotics and heavy metals. Bacteria in the biofilm structure are more resistant to stress factors than planktonic cells. Bacteria produce different signalling molecules, whose accumulation depends on cell density. Quorum sensing is a type of communication used to recognize the population density of the same species, which probably participates in biofilm formation. Thus, the presence of toxic pollutants in the soil can disrupt biofilm formation by inhibiting cell growth, specific gene expression, chemical signal compound production and exopolysaccharide formation^58–60^.

**Figure 3:**
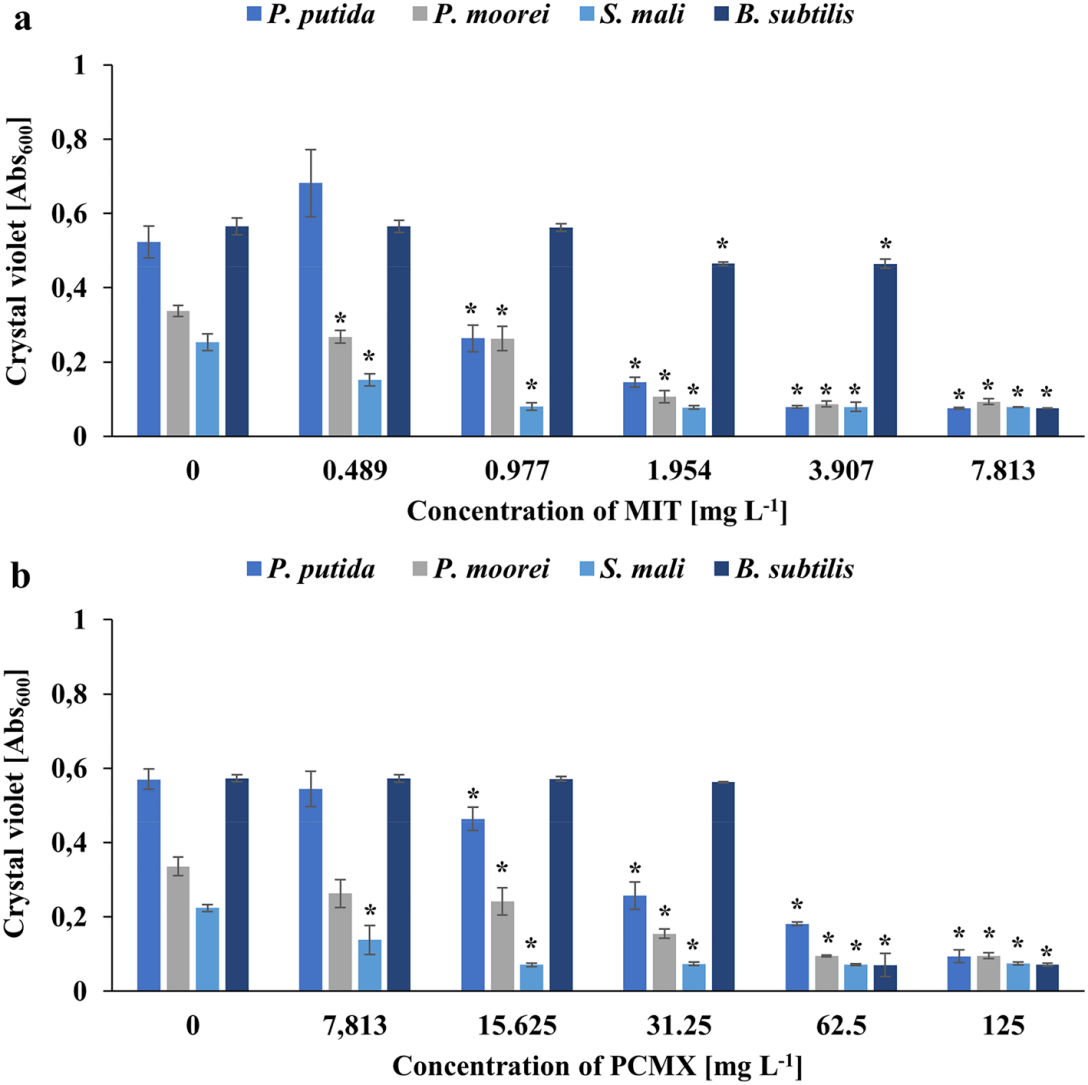
Effect of MIT (**a**) and PCMX (**b**) on biofilm formation by the tested strains. Data are expressed as the average of crystal violet absorbance ± SD (n = 4). The presented results were analysed using the Mann‒Whitney U test with *p < 0.05.

In the next step of this study, we compared the effects of MIT and PCMX on the tested soil bacterial strains using crystal violet in the microtiter plate assay (MPA). The inhibition of biofilm formation was measured by comparing biotic controls to tested samples supplemented with xenobiotics at concentrations of 0.489, 0.977, 1.954, 3.807, and 7.813 mg L^−1^ (MIT) and 7.813, 15.625, 31.25, 62.5 and 125 mg L^−1^ (PCMX). According to the study of Mathur et al., tested microorganisms were classified as high (*P. putida*, *P. moorei* and *B. subtilis*) and moderate (*S. mali*) biofilm producers^61^. A limited ability of the tested strains to form biofilms was observed in a dose-related manner with statistical significance (Fig. 3). For instance, the absorbance value in the biotic control of *P. putida* reached approximately 0.52, whereas the addition of MIT at a concentration of 0.977 mg L^−1^ reduced it to 0.26. In the samples with the addition of PCMX, a significant impact on biofilm formation was observed at a concentration of 15.625 mg L^−1^. The biofilm formation ability of *P. moorei* was significantly inhibited by even the lowest concentration of MIT. Cells of *P. moorei* treated with PCMX at a concentration of 15.625 mg L^−1^ showed a decrease in biofilm formation. Moderate inhibition of biofilm production by *B. subtilis* was noted after the addition of MIT at a concentration of 1.954 mg L^−1^. The *Bacillus* strain proved highly resistant to MIT, and a concentration as low as 7.813 mg L^−1^ caused an 80% decrease in absorbance. Likewise, high concentrations of PCMX did not inhibit biofilm formation by *B. subtilis*. Significant abolition of the ability to form biofilms was observed in the *B. subtilis* samples supplemented with PCMX at a concentration of 62.5 mg L^−1^.

The use of reclaimed water or sewage sludge in agriculture, without the control of the concentration of micropollutants, could have a negative effect on the capability of bacteria to form biofilms on plant surfaces and subsequently lead to decreased protection of plants. Soil exposure to MIT and PCMX may disrupt rhizosphere ecosystems and make plants more susceptible to disease, resulting in poor growth and decreased agricultural yield^62^.

### Reactive oxygen species

**Figure 4:**
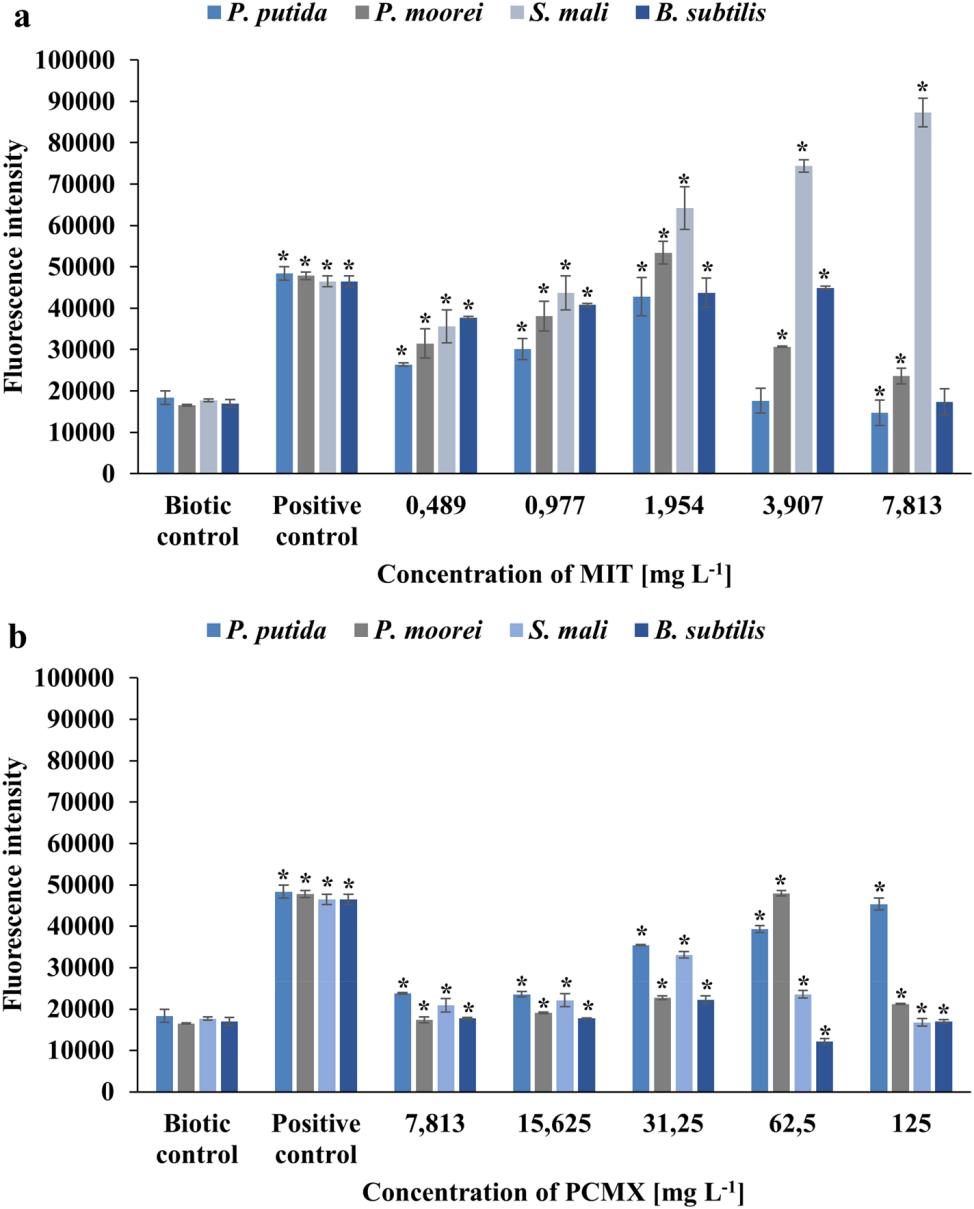
Fluorescence intensity representing the amount of intracellular ROS production in bacterial strains after their treatment with MIT (**a**) or PCMX (**b**). Data are expressed as the average fluorescence intensity ± SD (n = 3). The presented results were analysed using the Mann‒Whitney U test with *p < 0.05.

Bacterial cells that have been exposed to oxidants, such as biocides, have mechanisms for recognizing and eliminating them. ROS are harmful species that react with many biological components, causing them to malfunction. Excess ROS generation occurs when bacterial cells are subjected to environmental stress, resulting in the initiation of cell necrosis or apoptosis by damage to nucleic acids and proteins or disturbed cell membrane integrity^63,64^. Therefore, to determine the presence of ROS in cells treated with MIT or PCMX, the intensity of 2′,7′-dichlorofluorescein (DCF) fluorescence was measured. As shown in Fig. 4, the ROS intensity in the positive control in comparison to the biotic control was significantly higher (more than 2.5 times). Strains treated with MIT or PCMX showed higher ROS levels than untreated cells. Regardless of the strain or type of xenobiotic, the ROS levels rose with increasing biocide concentrations until they reached the MIC. Despite the high concentrations of xenobiotics, the ROS levels decreased, which might have resulted from bacterial cell death caused by oxidative damage. Sharma et al. have also reported similar results for gram-negative *Pseudomonas fluorescens* and gram-positive *B. subtilis* exposed to copper nanoparticles. The authors observed a decrease in the ROS level above MICs and suggested that the reason for cell death was caused by higher oxidative stress at minimal bactericidal concentrations^65^. The maximum ROS intensity induced by MIT was 4.93 times higher than that of the biotic control and was observed in *S. mali* at 7.813 mg L^−1^ MIT. On the other hand, the maximum intensity induced by PCMX was observed in *P. putida* at a concentration of 125 mg L^−1^ and was 3.1 times higher than that of the control. The higher increase in fluorescence under all treatment concentrations of MIT confirmed that MIT is more toxic to the tested bacteria than PCMX. The obtained results acknowledge the induction of ROS generation in response to the administration of MIT and PCMX as a mechanism of killing bacteria.

The formation of ROS is a key measure for assessing oxidative stress. Toxicity studies of numerous environmental contaminations, at an angle of oxidative stress, have emerged as critical biomarkers for assessing the environmental impact of pollution. Matejczyk et al., evaluated the toxicity of diclofenac and its metabolites, mixed with various compounds, on *E. coli* cells in WWTPs. The occurrence of xenobiotics in the growth environment of bacteria led to the induction of oxidative stress and intensified ROS generation^66^. Zheng et al., identified the influence of perfluoroalkyl substances on damage caused by ROS in the soil microorganism *B. subtilis*. The obtained results showed that with increasing concentrations of toxic substances, a decrease in the level of ROS was observed. Moreover, the activities of superoxide dismutase and catalase, which are antioxidant enzymes involved in the antioxidant defence system, were analysed. The authors observed the opposite trend of enzyme activity to ROS levels and suggested that it might be a result of stability between the oxidant system and antioxidant system in microorganism cells^67^.

### The influence of MIT and PCMX on the production of phytohormones

Soil beneficial bacteria are known to synthesize phytohormones, which may affect the growth and health of plants. Auxins are phytohormones produced by soil bacteria, and their mechanisms of action involve controlling the individual stage of plant growth by stimulating of cell division and elongation, differentiating of tissue, and assisting in apical dominance. Among known auxins, indole-3-acetic acid is the most abundant hormone produced by beneficial soil bacteria. *Pseudomonas, Bacillus, Rhizobium, Enterobacter, Micrococcus, Azospirillum, Actinomycetes*, and *Kocuria* are popular strains of PGPR capable of producing IAA and other auxins^68,69^. The effectiveness of microbial stimulation of plant growth may be dependent in part on changes in the production of IAA caused by toxic substances occurring in the growth environment. To evaluate of the influence of different concentrations of MIT or PCMX on the production of auxin, the tested bacteria were incubated on nutrient broth (NB) or Mueller–Hinton broth (MHB) medium supplemented with l-tryptophan as a precursor. To quantify IAA production, the most common spectrophotometric method based on the colour reaction of the Salkowski reagent with auxins was used. The concentration of IAA was calculated on the basis of the standard curve after 48 h of incubation, when the highest concentration IAA was observed. Among untreated bacteria, *P. moorei* produced a maximum amount of 54.30 ± 1.61 mg L^−1^ of IAA. The next strain able to produce a high amount of IAA was *P. putida*, with an average value of 38.86 ± 1.18 mg L^−1^. In the present study, neither *S. mali* nor *B. subtilis* was able to produce a high amount of phytohormones. The average concentration of IAA in the control samples was below 2 mg L^−1^. After exposure of *P. putida, P. moorei, S. mali*, and *B. subtilis* to 0.489, 0.977, and 1.954 mg L^−1^ MIT, IAA production decreased in a dose-dependent manner (Table 1). Taking into consideration the production of auxin at a mean concentration (0.977 mg L^−1^) of methylisothiazolinone by four bacterial strains, the maximum inhibition of IAA production (94.5%) was noted for *P. moorei*, followed by *S. mali* (67.2%) and *P. putida* (58.21%) in comparison to the control. Chloroxylenol exhibited less toxicity than MIT towards the tested strains. *P. putida* was able to secrete a larger amount of IAA with higher concentrations of the xenobiotic than in the control sample. IAA production was significantly inhibited in the case of the rest of the microorganisms, and PCMX at a mean concentration of 31.25 mg L^−1^ reduced IAA synthesis by 27.72, 100, and 100% for *P. moorei, S. mali,* and *B. subtilis*, respectively. The reduction in the synthesis of phytohormones at increasing concentrations of biocides could have been due to slower growth and disturbed physiological activity of microbial cells.

**Table 1:** Production of IAA by tested bacterial strains supplemented with MIT or PCMX after 48 h of incubation.

Treatment	Concentration [mg L^−1^]	Concentration of IAA [mg L^−1^]			
		*P. putida*	*P. moorei*	*S. mali*	*B. subtilis*
Control	0	38.86 ± 1.18	54.30 ± 1.61	1.71 ± 0.32	1.18 ± 0.19
MIT	0.489	37.63 ± 2.22	33.05 ± 2.24*	0.93 ± 0.15*	1.55 ± 0.43
	0.977	16.24 ± 1.16*	2.95 ± 0.29*	0.56 ± 0.12*	1.28 ± 0.08
	1.954	3.75 ± 1.48*	2.60 ± 0.08*	0.08 ± 0.20*	0.82 ± 0.05*
PCMX	7.813	39.24 ± 1.95	44.23 ± 0.92*	0.30 ± 0.02*	1.37 ± 0.42
	31.25	41.15 ± 1.92*	39.25 ± 0.20*	0 ± 0.01*	0 ± 0.04*
	62.5	42.44 ± 1.05*	30.26 ± 1.75*	0 ± 0.07*	0 ± 0.004*

Data are expressed as the average IAA concentration ± SD (n = 4). The presented results were analysed using the Mann–Whitney U test with *p < 0.05.

Because the method is based on the use of the Salkowski reagent, which can give a nonspecific colour reactions with other similar indolic compounds and could provide inaccurate information about quantities of IAA^69^, we decided to additionally use liquid chromatography coupled with tandem mass spectrometry. This technique allowed for confirmation of the presence of the tested phytohormone in the study samples. Comparison of the mass spectra of the tested samples with that of the IAA standard confirmed the presence of indole-3-acetic acid. IAA obtained from the culture supernatants had a retention time of 1.12 min and produced a spectrum identical to that of standard IAA, with a parent ion *m/z* of 176 and fragments at *m/z* 158, 149, 130, 103 and 96.

### The influence of MIT and PCMX on cellular membrane modification

**Figure 5:**
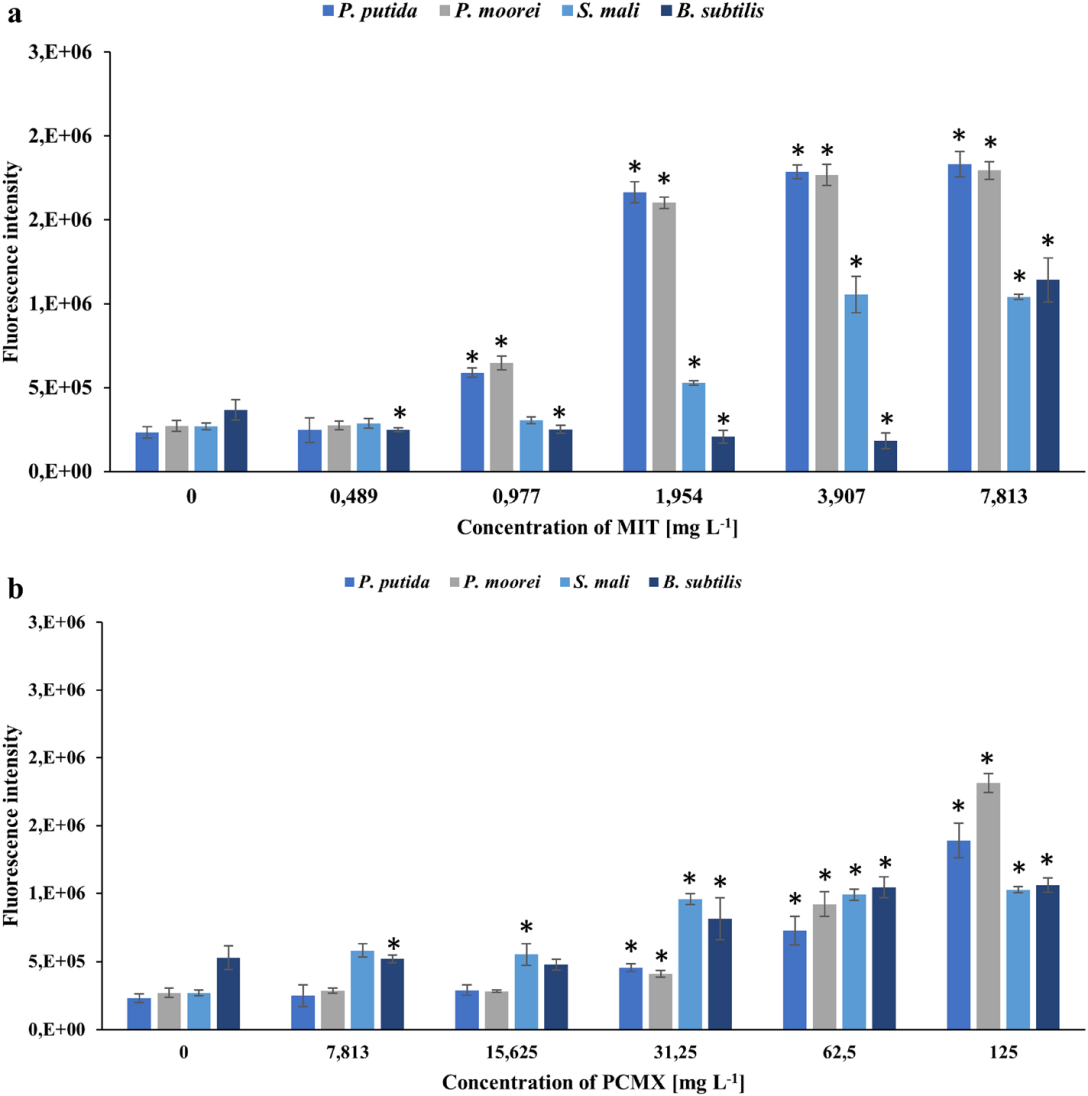
The effect of MIT (**a**) or PCMX (**b**) on the membrane permeabilization of the tested bacteria. Data are expressed as the average fluorescence intensity ± SD (n = 3). The presented results were analysed using the Mann‒Whitney U test with *p < 0.05.

The activity of many antibacterial agents is associated with the disturbance of bacterial cell membrane permeability. To investigate the possible mechanisms of the antibacterial activity of MIT and PCMX towards beneficial soil bacteria, their effect on the integrity of bacterial membranes was assessed with the fluorogenic dye SYTOX Green. This stain can penetrate only bacterial cells with the damaged plasma membrane and bind to nucleic acids, causing an increase in fluorescence intensity. We found that MIT and PCMX significantly changed the permeability of the cell membrane in all tested strains at concentrations above 0.489 mg L^−1^ and 15.625 mg L^−1^ for MIT and PCMX, respectively, compared to the biotic controls (Fig. 5). These results were closely correlated with bacterial growth inhibition by the tested preservatives.

Moreover, to confirm the effect of biocides on the components of cell membranes, the phospholipid (PL) profiles were evaluated. Phospholipids are the main components of the bacterial membrane, whose content is variable and depends on environmental conditions. The occurrence of toxic substances in the habitat of bacterial communities induces bacterial cells to adapt to potentially harmful surroundings modifying of membrane phospholipids for the regulation of membrane fluidity^70^.

**Figure 6:**
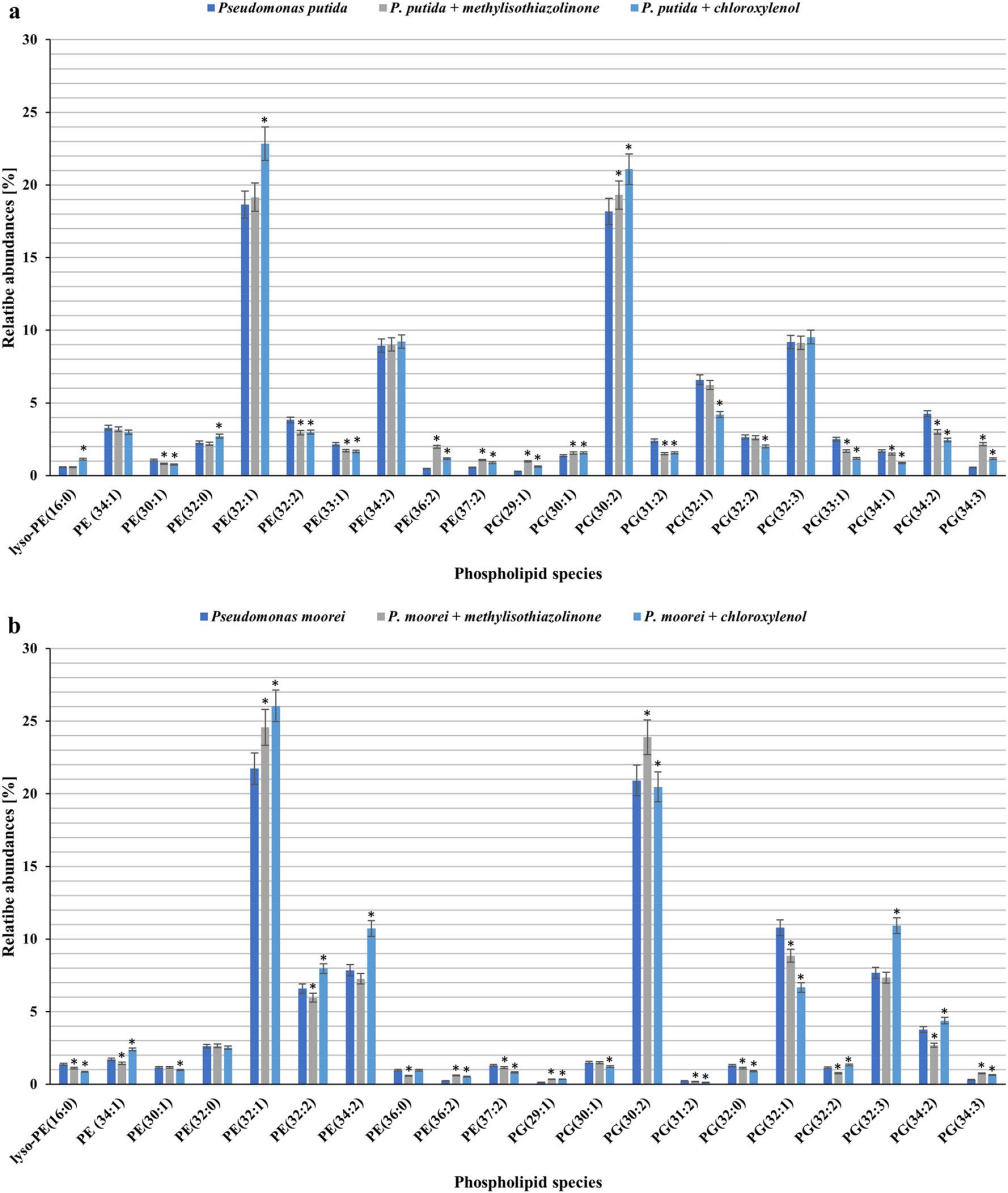

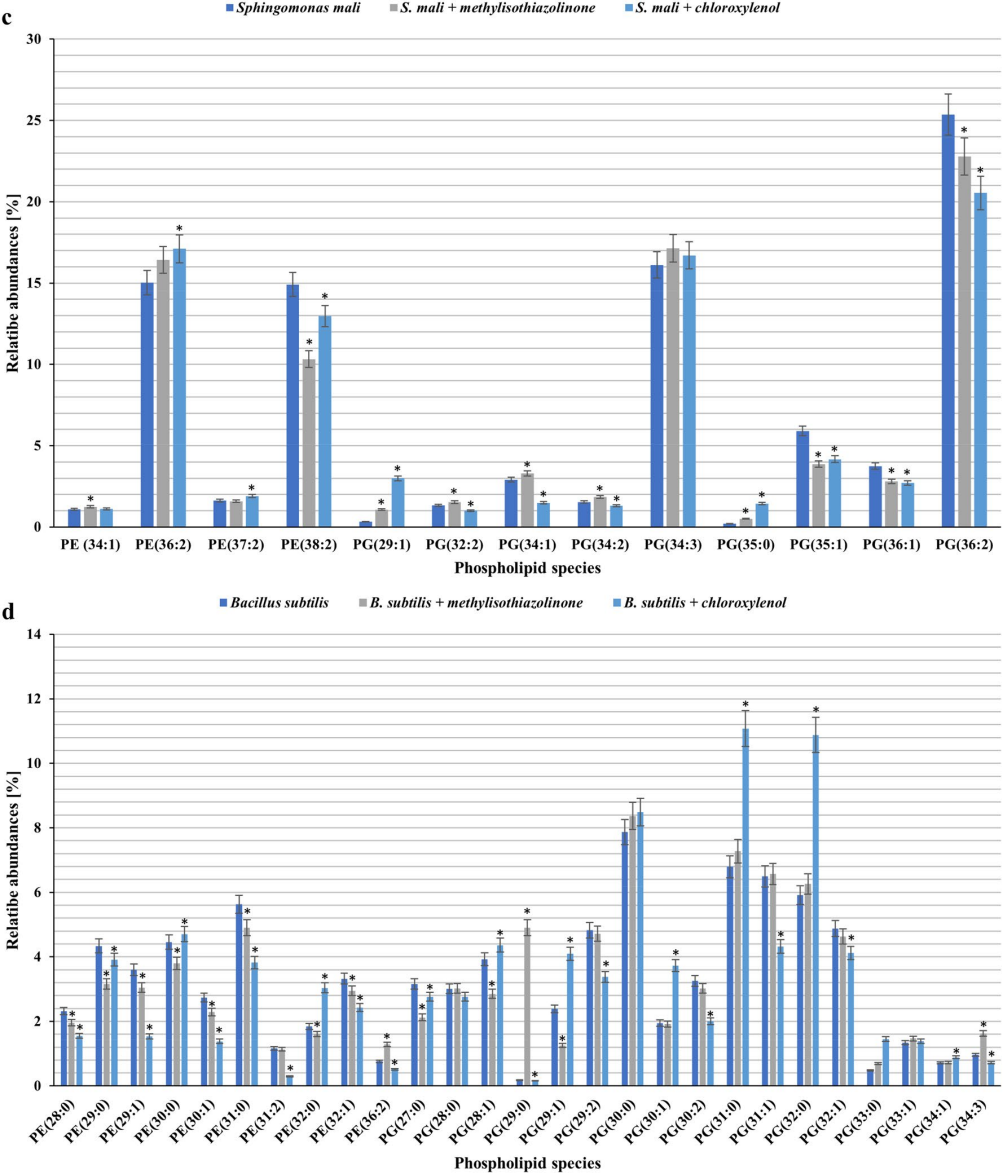
Changes in the contents of phospholipid molecular species determined in bacterial cells after incubation with MIT or PCMX [*P. putida* (**a**); *P. moorei* (**b**), *S. mali* (**c**), *B. subtilis* (**d**)]. Data are expressed as the average percentage ± SD (n = 3). The presented results were analysed using the Mann‒Whitney U test with *p < 0.05.

The changes in the PL profile were assessed by comparing the biotic control to the tested samples. Each bacterial strain was supplemented with biocides at a concentration below the MIC value, causing approximately 50% growth inhibition. Based on the mass spectra in negative ionization mode, the PL profile of each strain was identified. Despite high differentiation among microbial families, the bacterial membrane structure is highly conserved. Anionic phosphatidylglycerol (PG) and zwitterionic phosphatidylethanolamine (PE) are the major lipid elements of bacterial membranes^71,72^. Our results also indicated that the head groups of glycerophospholipids found in the control and treated samples were PE and PG. A comparison of the control samples with the treated cultures allowed us to observe the significant changes in the PL content only in the case of the *B. subtilis* cultures. After exposure to MIT, the amount of PE decreased to 31.67%, and the amount of PG increased to 66.5%. An analogous tendency was observed in the case of PCMX, but differences in the content of PE and PG between the tested samples and control were more significant (Table 2). Moreover, all the tested strains changed their PE/PG ratio after being supplemented with MIT or PCMX. Changes in the PE/PG ratio can help stabilize the membrane by reducing its fluidity. Murzyn et al.^73^ conducted computational analysis, which revealed that an increased quantity of PG, whose glycerol polar head is larger than that of ethanolamine, and a decrease in the PE content could lead to increased membrane stability and reduced fluidity. The modification of membrane composition as a process of adapting microorganisms to toxic contaminants is well documented^74–76^. Similar results were obtained by Bernat et al.^77^, where the influence of iturin A lipopeptide on the phospholipid composition of *B. subtilis* was determined. According to the literature, PE is a dominant phospholipid in the inner membrane of gram-negative bacteria, while the membranes of gram-positive species are enriched in anionic phospholipids^78^. Additionally, in our research, an increased amount of PE was observed in the gram-positive *B. subtilis* strain. Moreover, gram-positive bacterial membranes were found to be more susceptible to the applied biocides. A quantitative analysis of PL species was also conducted. For *P. putida,* *P. moorei, S. mali,* and *B. subtilis*, 10, 10, 4, and 10 PE species, and 11, 10, 9, and 17 PG species were identified. The alkyl chains (two per phospholipid species) of PE and PG in the respective strains consisted of 12–18 carbon atoms. The examined phospholipids included saturated, mono-unsaturated, and di-unsaturated fatty acids. In gram-negative bacteria, the dominant PE species were 32:1, 36:2, and 38:2, and the dominant PG species were 30:2, 34:3, and 36:2. In the case of gram-positive bacteria, it was difficult to indicate prevailing species of PE because of high differentiation and a similar amount of each species. The dominant species of PG were 30:0, 31:0, and 32:0. The detailed difference in the quantity of PL species is shown in Fig. 6.

**Table 2:** Changes in the total amount of PLs bacterial cells after incubation with MIT or PCMX.

Phospholipid classes	Sample		
	*P. putida*	*P. putida* + MIT [0.7 mg L^−1^]	*P. putida* + PCMX [62.5 mg L^−1^]
PE	44.08 ± 2.2	44.89 ± 2.24	47.4 ± 2.37
PG	53.59 ± 2.68	52.82 ± 2.64	48.94 ± 2.45
PE/PG	0.82 ± 0.0	0.85 ± 0.0*	0.97 ± 0.00*

	*P. moorei*	*P. moorei* + MIT [0.7 mg L^−1^]	*P. moorei* + PCMX [62.5 mg L^−1^]
PE	45.62 ± 2.28	46.64 ± 2.33	47.58 ± 2.38
PG	50.62 ± 2.53	49.94 ± 2.5	49.46 ± 2.47
PE/PG	0.90 ± 0.0	0.93 ± 0.0*	0.96 ± 0.0*

	*S. mali*	*S. mali* + MIT [1.4 mg L^−1^]	*S. mali* + PCMX [2.5 mg L^−1^]
PE	35.88 ± 1.79	35.87 ± 1.79	39.38 ± 1.97
PG	62.37 ± 3.12	60.13 ± 3.0	58.01 ± 2.9
PE/PG	0.58 ± 0.0	0.6 ± 0.0*	0.68 ± 0.0*

	*B. subtilis*	*B. subtilis* + MIT [5.0 mg L^−1^]	*B. subtilis* + PCMX [40.0 mg L^−1^]
PE	35.73 ± 1.79	31.67 ± 1.58*	27.71 ± 1.39*
PG	62.71 ± 3.14	66.65 ± 3.33	71.09 ± 3.55*
PE/PG	0.57 ± 0.0	0.48 ± 0.0*	0.39 ± 0.0*

Data are expressed as the average percentage ± SD (n = 3). The presented results were analysed using the Mann–Whitney U test with *p < 0.05.

Briefly, in *P. putida* and *P. moorei* cells treated with PCMX, a slight increase in the major forms of both PE and PG was observed (*P. putida*: PE 32:1, PG 30:2; *P. moorei*: PE 32:1, PE 32:2, PE 34:2, PG 30:2. PG 32:1, PG 32:3). Upon addition of MIT to *Pseudomanas* cultures, a slight increase was observed in PG 30:2 (*P. putida*) and PE 32:1, PG 30:2 (*P. moorei).* Moreover, a slight decrease was observed in the main PL species of *P. moorei* (PE 32:2, PG 32:1). In *S. mali* cells after exposure to MIT, both PE (38:2) and PG 36:2 levels increased. However, the presence of PCMX in the growth medium led to an increase in the amount of PE 36:2 and a decrease in PE 38:2 and PG 36:2. The changes in levels in phospholipid species are characteristic of microorganisms exposed to xenobiotics such as antimicrobial compounds, organotin compounds, pharmaceuticals, steroids and alcohols^79–82^. The study conducted by Simon et al. showed that *P. putida* KT2440 exposed to n-butanol results in changes in PL species levels. The authors observed a small increase in the principal forms of both PG and PE after adding 0.5 g L^−1^ butanol. In contrast, the abundance of PE and PG was reduced at a greater butanol concentration of 3 g L^−1^^81^. Additionally, Bernat et al.^79^ noted the modification of the PL species profile in *Pseudomonas* sp. B-219 exposed to tributyltin.

## Conclusion

In the present study, for the first time, disinfectant agents (MIT and PCMX) were tested against four soil bacteria, *P. putida, P. moorei, S. mali* and *B. subtilis*. The effects of the different concentrations of the studied compounds on the growth, viability, biofilm formation, ROS generation, IAA production and cell membrane modification of the tested strains were investigated. The results indicated that MIT and PCMX at increasing concentrations inhibited the growth of bacteria. MIT appeared to be more toxic to the tested organisms than PCMX. However, the toxicity of preservatives against bacterial cells depended on the bacterial species. Furthermore, reduced viability and inhibition of biofilm formation were clearly observed with increasing concentrations of each biocide. A significant increase in DCF fluorescence intensity suggested that the antioxidant system of microorganism cells at increasing concentrations of xenobiotics was unable to eliminate excess ROS. In addition, MIT and PCMX altered the metabolic pathway and disturbed the synthesis of the phytohormone indole-3-acetic acid. The presence of MIT and PCMX in the growth environment of bacteria resulted in changes in the phospholipid composition and increased cell membrane permeability. The ecotoxicity assessment conducted in this study indicated that MIT and PCMX pose a risk to soil bacteria. Due to the high use of personal care products and household chemicals that are preserved by MIT and PCMX, their unregulated discharge in the soil and aquatic environments may impact the useful bacterial population and the health of the natural environment.

Personal care products, among other preservatives, such as MIT and PCMX, have harmful effects on nontarget organisms. In the near future, continuous monitoring and measures of biocide levels must be undertaken and would be helpful for controlling and predicting their ecotoxic effects on the environment. Moreover, the ecotoxicological risks of MIT and PCMX to different terrestrial organisms need to be evaluated because of limited data in this field. Analysis of the influence of single compounds, as well as a mixture of these contaminants, is also needed. Finally, in vitro studies focus only on certain ecotoxicological mechanisms and aspects, without taking into account the effect on the populations of organisms and the ecosystem. Therefore, the toxicity activity of these preservatives needs to be analysed in in situ, in vivo, and in silico studies, such as towards microbial communities.

## Materials and method

### Bacterial cultures and chemicals

*Pseudomonas putida* (DSM 291), *Pseudomonas moorei* (DSM 12647), *Sphingomonas mali* (DSM 10565) and *Bacillus subtilis* (DSM 3657) were obtained from the German Collection of Microorganisms and Cell Cultures GmbH (Germany). Methylisothiazolinone, chloroxylenol, 3-indoleacetic acid, iron(III) chloride, phospholipid standards and ammonium format were purchased from Merck (Poland). SYTOX™ Green Nucleic Acid Stain, General Oxidative Stress Indicator (CM-H_2_DCFDA, 5-(and-6)-chloromethyl-2′,7′-dichlorodihydrofluorescein diacetate, acetyl ester) and AlamarBlue Cell Viability Reagent were obtained from Thermo Fisher Scientific (Poland). Difco™ nutrient broth and Mueller Hinton broth were purchased from Becton Dickinson (Poland). DMSO and phosphate-buffered saline (PBS) were obtained from BioShop (Canada). Methanol, ethyl acetate, hydrochloric acid, acetic acid, crystal violet and sodium sulfate anhydrous were obtained from Chempur (Poland). All of the other reagents with a high analytical purity grade were purchased from Avantor (Poland).

### Determination of bacterial sensitivity to MIT and PCMX

The toxicity potential of MIT and PCMX was determined using pure bacterial cultures according to the standard microdilution method of the Clinical and Laboratory Standards Institute^83^. All experiments were carried out in nutrient broth for *P. putida*, *P. moorei* and *S. mali* and Mueller Hinton broth for *B. subtilis*. The bacterial sensitivity towards MIT and PCMX was assessed over the concentration range from 0.0305625 to 31.25 mg L^−1^ and 0.977 to 1000 mg L^−1^ with two drug dilution steps. Stock solutions of MIT (62.5 µg mL^−1^) and PCMX (2 mg mL^−1^) were prepared in ethanol and diluted in adequate growth medium. The quantity of ethanol in the tested samples was below 5 µL, and toxic effects on the microorganisms were not observed. The growth of soil bacteria was carried out in 96-well cell culture plates by adding 100 µL suitable growth medium with or without the tested compounds and 100 µL bacterial inoculum prepared in NB or MHB to each of the tested wells. The final density of the tested strains was 1 × 10^6^ CFU mL^−1^. Simultaneously, the tested samples were led controls, abiotic controls, without the addition of microorganisms and with the addition of xenobiotics, biotic controls with the addition of microorganisms and without the addition of xenobiotics and controls of nonsupplemented medium. Adequate controls and tested samples were incubated for 24 h (*P. putida*, *P. moorei*) and 48 h (*S. mali* and *B. subtilis*) at 28 °C. The minimum inhibitory concentration (MIC) and bacterial susceptibility were determined based on optical density (λ = 630 nm) using a Multiskan TM FC Microplate Photometer spectrophotometer (Thermo Fisher Scientific). The results of the antibacterial effect of MIT and PCMX are shown as a percentage of the control samples, and MIC values are expressed in mg L^-1^.

### Determination of bacterial cell viability by AlamarBlue® assay

The AlamarBlue® dye used for the assessment of the viability of bacterial cells after incubation with MIT and PCMX was performed in accordance with the manufacturer’s protocol and literature data^80^. Ten microliters of a fluorescent dye was added to the 96-well plates prepared as in sensitivity testing over the concentration range from 0.489 to 7.813 mg L^−1^ (MIT) and 7.813 to 125 mg L^−1^ (PCMX). The adequate controls were made up the same as for the growth inhibition test with the addition of 10 µL dye. Then, the cells were incubated at a temperature of 28 °C for 3 h. The reduction of resazurin to resorufin was evaluated by fluorescent measurement at λ = 540 nm using a multimode microplate reader BMG LabTech FLUOstar Omega (BMG LABTECH GmbH, Germany). The results are shown as a percentage of the control sample.

### Measurement of biofilm formation with a microtiter plate assay

The microtiter plate assay was carried out as previously described by Lee et al.^84^. Clear polystyrene 96-well plates with a flat bottom and untreated surface (Thermo Fisher Scientific, Poland) were prepared in the same way as in sensitivity testing at the concentration range from 0.489 to 7.813 mg L^−1^ (MIT) and 7.813 to 125 mg L^−1^ (PCMX) and with the biotic, abiotic and medium controls. After incubation and optical density measurements, media and unattached cells were removed. Next, the wells were washed twice with 0.85% NaCl, and 96% ethanol was added for 20 min for the fixation of sedentary cells. After removing ethanol, the plates were air-dried at room temperature. Crystal violet solution (0.1%) was added to each well to stain the bacterial biomass. After 30 min of incubation, the solution was removed, and the wells were washed three times with saline solution. Finally, 33% acetic acid was added to the air-dried wells to completely dissolve crystal violet, and to obtain homogenized solution, the plates were shaken on a rotary shaker. The absorbance was measured at 600 nm by a microplate reader (FLUOstar Omega).

### Intracellular ROS detection

The intracellular reactive oxygen species production in bacterial cells, untreated and treated with the preservatives, was measured with the use of the nonfluorescent CM-H_2_DCFDA compound according to the manufacturer’s instructions and the study of Zawadzka et al.^85^. Briefly, after incubation, each bacterial inoculum was centrifuged at 10,174 × g for 5 min, washed with PBS three times, and resuspended in PBS. Then, the bacterial cells together with different concentrations of the tested compounds (0.489–7.813 mg L^−1^ for MIT and 7.813–125 mg L^−1^ for PCMX) were transferred to a black 96-well plate. H2O2 was used as a positive control at a concentration of 70 µM. Moreover, biotic and abiotic controls were prepared. The plates were incubated in the dark for 15 min at 28 °C. Next, CM-H_2_DCFDA solution at a final concentration of 5 µM was added to each well, and the plates were incubated in the dark for 30 min at 37 °C. ROS fluorescence was measured using a FLUOstar Omega reader with an excitation/emission wavelength of 495/520 nm.

### Effect of xenobiotics on IAA production

The effect of MIT and PCMX on IAA production by bacterial cultures was determined by the modified method of Maheshwari et al.^86^. The bacterial inoculum after 24 or 48 h of incubation on NB or MHB medium was transferred to fresh NB or MHB broth supplemented with L-tryptophan (0.15%) and the xenobiotics at concentrations of 0.489, 0.977, and 1.954 mg L^−1^ for MIT and 7.813, 31.25, and 62.5 mg L^−1^ for PCMX in a 50-mL Erlenmeyer flask. The biotic and abiotic controls and treated cells were incubated statically at 28 °C for 72 h. The tested samples and controls were measured every 24 h. Following the incubation, 1.5 mL of each sample was centrifuged at 10,174 × g for 10 min, and the clear supernatant was mixed with an equal volume of Salkowski reagent (1 mL of 0.5 M FeCl_3_ in 49 mL of 35% HClO_4_). The mixture was incubated in the dark for 30 min. After that, a quantity of IAA in the sample was measured at λ = 523 nm using the spectrophotometer SPECORD 200 (Analytic Jena, Germany) against a standard graph of pure IAA.

To eliminate false-positive results from the Salkowski assay, LC–MS/MS qualitative analyses for the verification of auxin production were conducted. The samples for qualitative analysis were prepared in the same manner as for the Salkowski method. After incubation, the whole volume of the samples was centrifuged at 2900 × g for 15 min, and the supernatants were acidified to pH 3 with HCl and shaken for 10 min with ethyl acetate (1:1). The collected organic phase was dried with anhydrous Na_2_SO_4_ and evaporated to dryness under reduced pressure at 40 °C. The residues were dissolved in 1 mL ultrapure methanol and then diluted with 2% acetonitrile with the addition of 0.1% formic acid. The detection of IAA was performed on an MS/MS system (4500 QTRAP spectrometer (SCIEX, USA) coupled with a microLC 200 system (Eksigent, USA). The chromatographic separation was carried out on an Eksigent C18 (0.5 mm × 50 mm × 3 mm, 120 Å) column at 50 °C. The mobile phases for the LC–MS/MS analysis consisted of water supplemented with 0.1% formic acid (phase A) and acetonitrile supplemented with 0.1% formic acid (phase B). The gradient profile was as follows: 98% A until 0.2 min, a linear increase to 98% B in 4 min, maintained until 6 min, returned to the initial conditions from 6 to 6.2 min and held until 6.7 min with a constant flow rate of the mobile phases at 35 μL min^−1^. The injection volume was 10 µL. The MS/MS scanning based on the information-dependent acquisition (IDA) method included multiple reaction monitoring (MRM) pairs and enhanced product ion (EPI) scans in positive ionization mode. IAA was detected based on m/z 176 > 130 (DP: 46, CE: 23) and 176 > 103 (DP: 46, CE: 33) ions. EPI scans worked at m/z 50–200, and mass spectra were collected for the identification of auxin presence. The microESI ion source was used to carry out analyses with set parameters: CUR (25), IS (4500), GS1 (30), GS2 (20), and TEM (400). Data analysis was carried out with Analyst™ software version 1.6.2 (SCIEX, USA).

### Evaluation of bacterial membrane permeabilization by SYTOX Green assay

The SYTOX Green assay was used to evaluate the bacterial cell membrane permeability according to the method described by Felczak et al.^87^ with modifications. SYTOX Green penetrates the damaged cell membrane and binds to DNA, giving an intense green fluorescence. Bacterial cultures, with and without the addition of xenobiotics, were prepared as in sensitivity testing at concentrations ranging from 0.489 to 7.813 mg L^−1^ (MIT) and 7.813 to 125 mg L^−1^ (PCMX) Adequate controls were also prepared (biotic, abiotic and control of medium). After incubation, bacterial suspensions were stained with 4 µL SYTOX Green dye (50 µM) for 15 min in the dark. Subsequently, the fluorescence of the DNA-bound dye was detected by a FLUOstar OMEGA reader with excitation and emission wavelengths of 485/535 nm. The values are represented as the percentage of the biotic control.

### Bacterial phospholipid analysis by HPLC–MS/MS

Phospholipids of the tested bacterial strains were extracted according to a previous method^80^ with modifications. Bacterial cultures were incubated in Erlenmeyer flasks (100 mL) on NB or MHB medium supplemented with MIT or PCMX at different concentrations. The final bacterial density was 1 × 10^6^ CFU mL^−1^. Adequate control wells without the addition of xenobiotics (biotic control) were prepared. After 24 or 48 h of incubation at 28 °C, bacterial biomass was separated by centrifugation at 5723 × g. It was disintegrated with 5 mL methanol and a glass matrix Ø 0.1 mm on a Mixer Mill MM400 (Retsch, Germany). Next, the homogenate was centrifuged (5723 × g, 10 min), and the supernatant was vortexed with 10 mL chloroform and 1 mL 0.85% NaCl for 4 min. The lower organic phase was collected, hydrogenated, and evaporated. The obtained phospholipid extracts were dissolved in 1 mL methanol. The lipid content was determined according to the method described by Zawadzka et al.^80^ with the use of an Agilent 1200 HPLC (Agilent, USA) and a 4500 QTRAP mass spectrometer (SCIEX, USA) with an ESI source.

### Statistical analysis

The obtained data are expressed as the mean ± SD. The nonparametric Mann–Whitney U test, with values of *p < 0.05 to estimate the statistical significance, was conducted to compare the treated population with the control. The statistical analyses were carried out using TIBCO Statistica™ 13.3 (StatSoft Poland 2017) and Excel, Microsoft 365 Business (Microsoft Corporation, USA).

## Data Availability

The data presented in this study are available on request from the corresponding author.

## Abbreviations

AB: AlamarBlue
ATP: Adenosine triphosphate
CE: Collision energy
CUR: Curtain gas
DP: Declustering potential
EPI: Enhanced product ion
ESI: Electrospray ionization
GS: Gas
HPLC: High-performance liquid chromatography
IAA: Indole-3-acetic acid
IDA: Information-dependent acquisition
IS: Ion source
LC‒MS/MS: Liquid chromatography-tandem mass spectrometry
MHB: Mueller–Hinton broth
MIC: Minimal inhibitory concentration
MIT: Methylisothiazolinone
MRM: Multiple reaction monitoring
m/z: Mass-to-charge ratio
NB: Nutrient broth
PBS: Phosphate buffer saline
PCP: Personal care products
PCMX: Chloroxylenol
PE: Phosphatidylethanolamine
PG: Phosphatidylglycerol
PGPR: Plant growth-promoting rhizobacteria
PL: Phospholipid
ROS: Reactive oxygen species
SARS-COV-2: Severe acute respiratory syndrome coronavirus 2
SD: Standard deviation
TEM: Temperature
WWTP: Wastewater treatment plant
